# From Minor Knee Laceration to Multisystem Catastrophe: A Five-Year-Old Boy's Battle With Protein C Deficiency and Purpura Fulminans

**DOI:** 10.7759/cureus.111937

**Published:** 2026-07-02

**Authors:** Sara Ali, Sofia Alfalasi, Mohammed Al Nuaimi

**Affiliations:** 1 Department of Pediatrics, Sheikh Shakbout Medical City (SSMC), Abu Dhabi, ARE; 2 Division of Pediatric Hematology and Oncology, Department of Pediatrics, Sheikh Khalifa Medical City, Abu Dhabi, ARE; 3 Department of Pediatrics, College of Medicine and Health Sciences, United Arab Emirates University, Al Ain, ARE

**Keywords:** acquired coagulopathy, pediatric thrombosis, protein c deficiency, purpura fulminans, skin necrosis

## Abstract

Purpura fulminans (PF) is a rapidly progressive thrombotic emergency. Trauma-induced acquired protein C deficiency is exceptionally rare in pediatric patients. This case adds to the limited literature describing PF triggered by minor traumatic injury, complicated by delayed recognition and resource limitations.

A previously healthy five-year-old boy developed rapidly expanding purpura and skin necrosis after a knee laceration from flying debris in Gaza. Lesions progressed to involve the feet, groin, thighs, back, and upper arm, ultimately resulting in 14.5% total body surface area (TBSA) necrosis and right above-knee amputation. Initial workup showed low protein C and extensive microvascular thrombosis, consistent with trauma-associated PF. Management included unfractionated heparin infusion guided by anti-Xa levels, broad-spectrum antibiotics, serial debridements, and staged grafting. Protein C normalized after recovery, and genetic testing was negative. He completed one year of anticoagulation with no recurrence of thrombosis.

Early recognition, precise treatment, and multidisciplinary care are lifesaving in trauma-induced PF. Differentiating acquired from congenital protein C deficiency prevents unnecessary lifelong therapy.

## Introduction

Purpura fulminans (PF) is a rare but life-threatening thrombo-hemorrhagic emergency characterized by rapidly progressive purpuric skin lesions, microvascular thrombosis, and tissue necrosis. It often occurs alongside disseminated intravascular coagulation (DIC) and may progress rapidly to systemic complications, including shock, limb loss, and death. Therefore, PF is considered both a hematologic and dermatologic emergency requiring early recognition and urgent management [1].

In children, PF is most frequently linked to disruption of endogenous anticoagulant systems, particularly the protein C pathway. Protein C is a vitamin K-dependent anticoagulant enzyme that, once activated, downregulates thrombin generation through inactivation of clotting factors V and VIII. Beyond anticoagulation, activated protein C also exerts anti-inflammatory and endothelial protective effects [2,3].

PF is traditionally categorized into congenital and acquired forms. Congenital protein C deficiency, typically due to homozygous or compound heterozygous variants in the PROC gene, often presents in the neonatal period with severe coagulopathy and fulminant thrombosis, frequently requiring protein C replacement and long-term anticoagulation [4,5]. In contrast, acquired protein C deficiency is more commonly encountered beyond infancy and is usually triggered by severe infection, particularly meningococcemia and septic shock, where inflammation-mediated endothelial injury and consumption of protein C promote dermal vascular thrombosis [5-7]. Less commonly, non-infectious triggers such as trauma, surgery, malignancy, and autoimmune disease have been described, but pediatric evidence remains limited [8].

This case adds to the limited pediatric literature on trauma-associated PF and highlights the diagnostic complexity of interpreting protein C levels during acute illness, when transient reductions may mimic congenital deficiency.

## Case presentation

**Figure 1:**
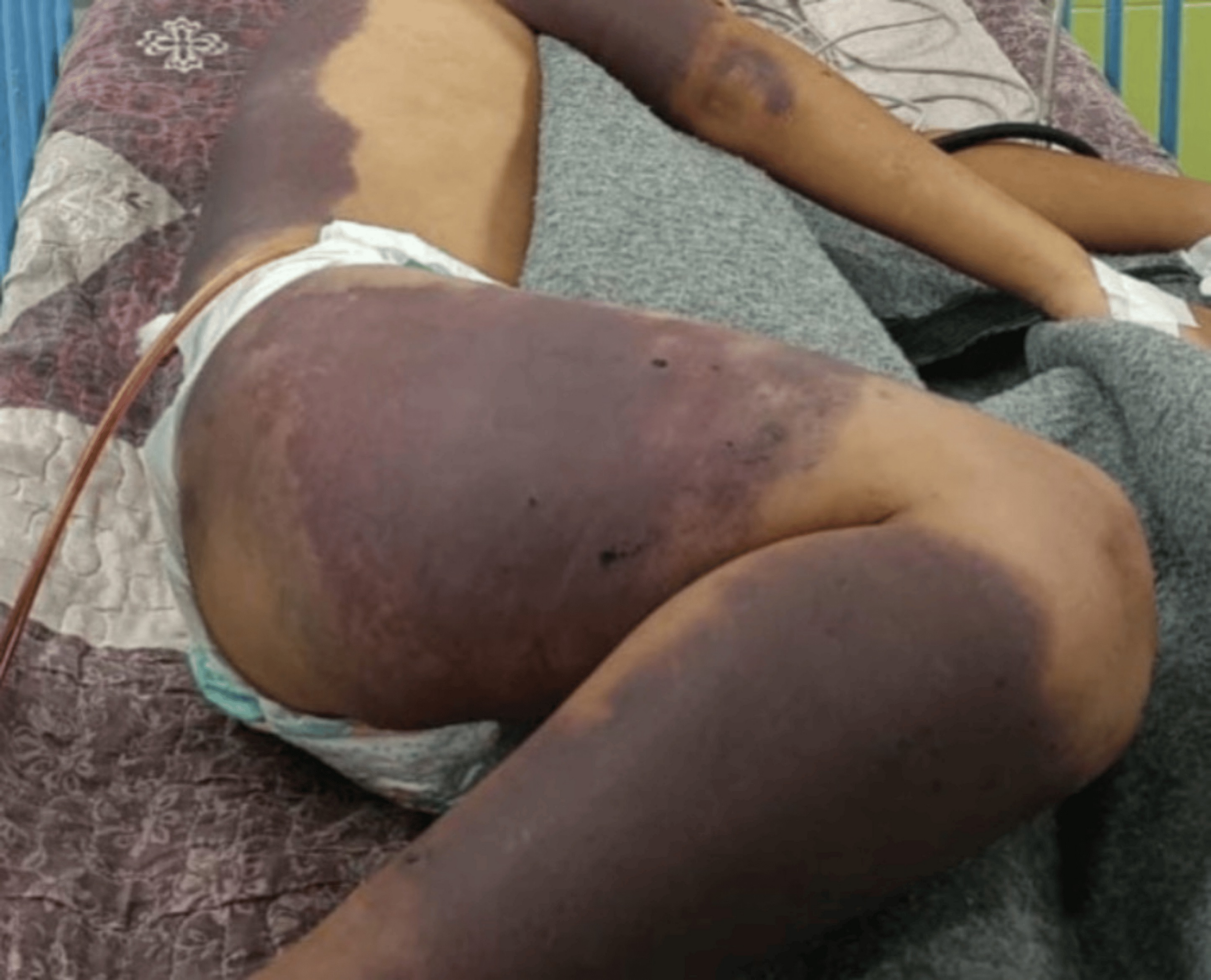
Extensive purpuric lesions involving the trunk, back, right upper limb, and bilateral thighs at initial presentation, prior to initiation of anticoagulation.

A previously healthy five-year-old boy from Gaza, Palestine, was transferred directly to our emergency department for advanced care following a blast event. The child had sustained minor trauma to the right lower limb after being struck by flying debris from rocket remains. Shortly thereafter, he developed rapidly evolving purpuric lesions over the feet and groin, which progressed to involve the trunk, back, shoulders, and upper and lower limbs (Figure 1).

During initial management in Gaza, anticoagulation was started with enoxaparin, with transient clinical improvement. Due to limited availability of parenteral anticoagulants, therapy was subsequently transitioned to warfarin for approximately one week. Despite treatment, the condition deteriorated, progressing to compartment syndrome of the right lower limb requiring emergency fasciotomy and eventual above-knee amputation. He also reportedly received an additional dose of warfarin the day prior to medical evacuation.

Initial concerns were raised at the referring center regarding possible warfarin-induced skin necrosis contributing to worsening tissue injury. However, based on available clinical history and documentation, most tissue necrosis and thrombotic changes were already present prior to significant warfarin exposure, making warfarin-induced skin necrosis unlikely. The clinical course was more consistent with trauma-associated PF in the setting of severe acquired protein C deficiency.

**Figure 2:**
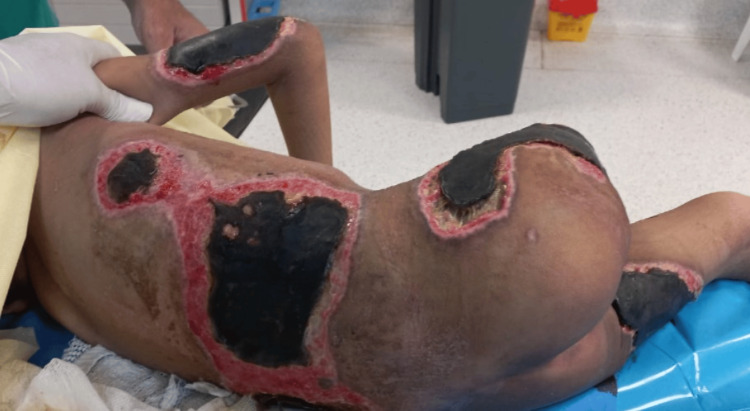
Multiple well-demarcated areas of cutaneous necrosis with black eschar formation and surrounding erythema developing after treatment/intervention.

**Figure 3:**
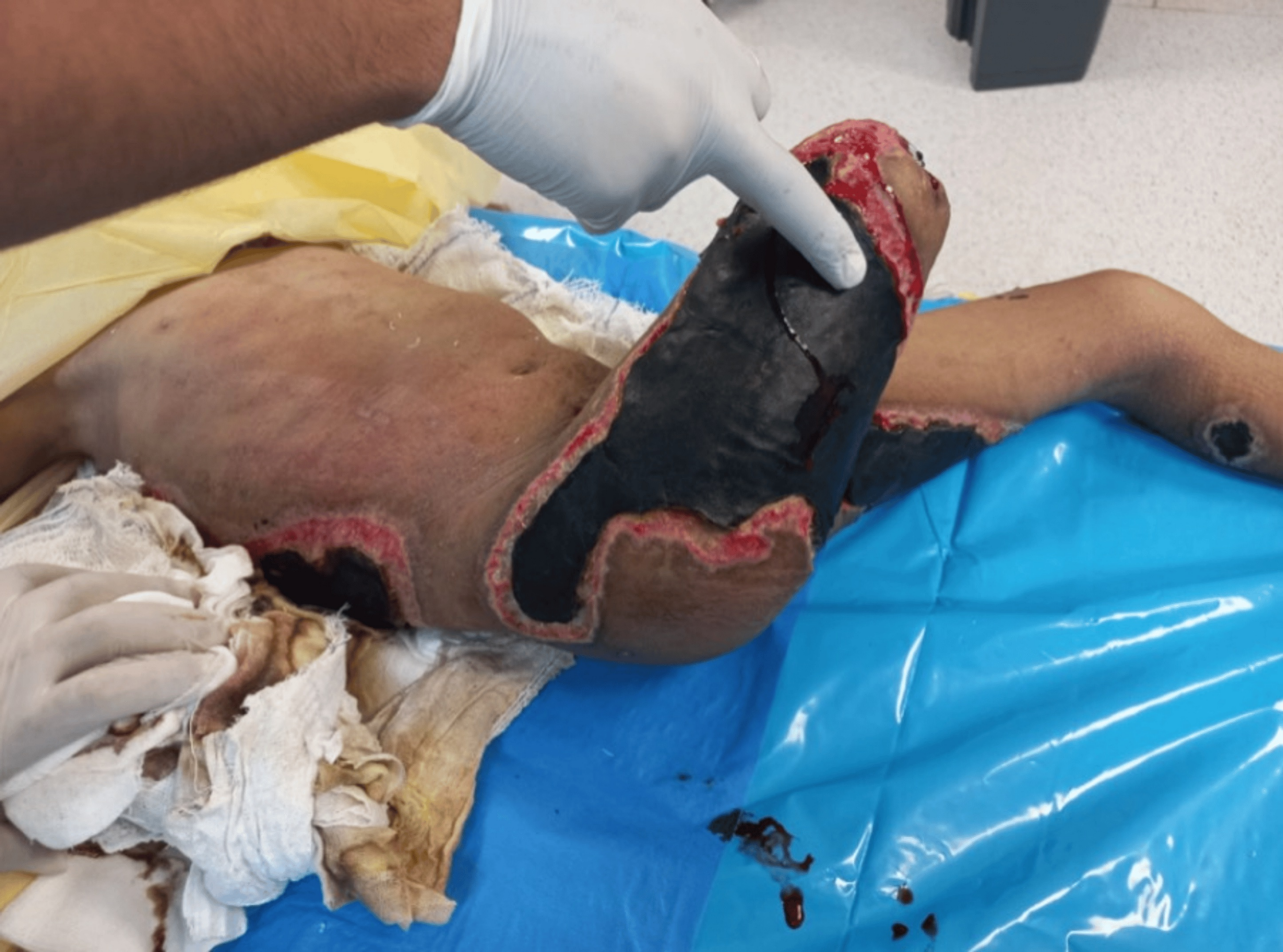
Right above-knee amputation stump with contralateral necrotic eschar following surgical intervention.

Upon arrival at our tertiary center, the patient appeared anxious but alert. Vital signs were: respiratory rate: 26 breaths/min, heart rate: 159 beats/min, oxygen saturation: 98% on room air, blood pressure: 108/65 mmHg, and temperature: 37.8°C. Physical examination revealed 14.5% total body surface area (TBSA) necrotic wounds involving the bilateral thighs, lower back, and right upper arm (Figure 2). A right above-knee amputation stump was present with healthy margins (Figure 3). No respiratory, cardiovascular, neurological, or abdominal abnormalities were noted.

Initial laboratory evaluation demonstrated reduced protein C activity (37%), protein S activity (66%), and protein S free antigen (77%). Coagulation studies showed prolonged prothrombin time (PT) of 17.4 s, International Normalized Ratio (INR) of 1.34, and activated partial thromboplastin time (aPTT) of 50.6 s, with elevated fibrinogen (5.05 g/L) and D-dimer (0.60 µg/mL). Antithrombin III activity was 89%. Platelet count was 150 ×10^9^/L. Inflammatory markers were significantly elevated, including C-reactive protein (CRP) (220 mg/L), with leukopenia (white blood cell count (WBC) 3.75 ×10^9^/L) and anemia (hemoglobin 96 g/L) (Table 1).

**Table 1:** Patient laboratory parameters compared with reference ranges. Hb, hemoglobin; WBC, white blood cell count; CRP, C-reactive protein; PT, prothrombin time; aPTT, activated partial thromboplastin time; INR, International Normalized Ratio

Blood Parameters	Patient Results	Reference Range
Hb (g/L)	96	115-145
WBC (x10⁹/L)	3.75	5.5-15.5
CRP (mg/L)	220	≤5.0
Platelets Count (x10⁹/L)	150	140-400
PT (seconds)	17.4	12.1-14.5
aPTT (seconds)	50.6	33.6-43.8
INR	1.34	0.92-1.14
Fibrinogen (g/L)	5.05	1.62-4.01
D-dimer (µg/mL)	0.60	≤0.50
Protein C activity (%)	37	70-140
Protein S activity (%)	66	72-126
Antithrombin III (%)	89%	80-120

The coagulation profile was consistent with an early or evolving consumptive coagulopathy in the setting of systemic inflammation, which may explain the relatively preserved platelet count and elevated fibrinogen rather than overt DIC.

Arterial and venous Doppler studies showed no evidence of ongoing thrombosis. Infectious evaluation and thrombophilia testing were performed as clinically indicated and did not reveal an alternative primary hypercoagulable disorder; however, interpretation was limited by acute inflammation and recent anticoagulation exposure.

At our center, he was managed with continuous unfractionated heparin infusion, broad-spectrum antimicrobial therapy (meropenem and linezolid), and serial wound debridements after stabilization. Repeat testing during recovery showed normalization of protein C activity (95%), and genetic testing for congenital protein C deficiency was negative, supporting an acquired etiology. The patient remained stable, continued anticoagulation therapy initially with enoxaparin and later rivaroxaban, and progressed through rehabilitation without further thrombotic complications.

## Discussion

PF reflects catastrophic dysregulation of hemostasis driven by widespread activation of coagulation pathways, endothelial injury, and depletion of natural anticoagulant proteins. Dermal small-vessel thrombosis is the central pathologic process, producing hemorrhagic purpura that may rapidly progress to necrosis [1,5].

Distinguishing congenital from acquired protein C deficiency is essential because short-term management and long-term outcomes differ. Congenital deficiency is typically severe, often presenting in the neonatal period with extensive thrombosis and DIC, and may require lifelong anticoagulation and/or replacement therapy [4,5]. In contrast, acquired deficiency results from consumption of protein C and other anticoagulant proteins during systemic inflammatory states. Clinical recovery is commonly accompanied by normalization of protein C levels, allowing discontinuation of anticoagulation in most cases without recurrence [5,7].

Trauma-associated PF is rare in children. Proposed mechanisms include endothelial disruption, inflammatory cytokine release, increased tissue factor exposure, and secondary consumption of anticoagulant proteins. Available evidence is largely case-based but suggests that trauma can precipitate PF even in previously healthy children [5,8]. This overlap may create diagnostic uncertainty early in the disease course, particularly when protein C testing is performed during the acute consumptive phase.

Interpretation of protein C and protein S levels during acute illness is challenging, as values may be transiently reduced due to inflammation, consumptive coagulopathy, hepatic dysfunction, and prior anticoagulant exposure. Vitamin K antagonists such as warfarin can further suppress protein C levels, owing to its short half-life, potentially contributing to warfarin-associated skin necrosis and complicating diagnostic interpretation. Accordingly, expert recommendations emphasize repeat testing after clinical stabilization and consideration of genetic testing to confirm congenital disease [5].

In the present case, reduced protein C and protein S levels were identified during the acute thrombotic phase. It is well established that natural anticoagulant levels may be transiently decreased during acute thrombosis due to consumption and inflammatory suppression. Importantly, serial follow-up demonstrated normalization of protein C activity (95% at four months and 85% at seven months), supporting a transient acquired deficiency rather than an inherited thrombophilia.

This case highlights that trauma-associated PF may mimic congenital protein C deficiency in the acute setting, particularly when anticoagulation with warfarin is introduced during evolving coagulopathy. Recognition of this transient pattern is essential to avoid misdiagnosis and unnecessary lifelong anticoagulation.

Management requires immediate supportive care, treatment of the underlying trigger, and correction of coagulopathy. In severe cases, protein C concentrate and anticoagulation have been reported to be beneficial in pediatric PF, although the evidence remains limited and largely retrospective [5,6]. Early recognition and timely intervention remain the strongest predictors of outcome, particularly in preventing progression to limb-threatening necrosis.

## Conclusions

This case underscores that trauma-associated PF may occur in previously healthy children and can mimic congenital protein C deficiency during the acute phase. Transient reductions in natural anticoagulants are commonly observed during acute thrombosis, systemic inflammation, and in the context of recent anticoagulation exposure, highlighting the importance of repeat testing after clinical stabilization. Accurate differentiation between acquired and congenital protein C deficiency is critical to guide appropriate management and avoid unnecessary long-term anticoagulation. However, interpretation of coagulation studies in the acute setting may be confounded by illness severity and therapeutic interventions, including warfarin exposure, and should therefore be undertaken with caution. Early recognition and prompt multidisciplinary supportive care remain essential to prevent progression to severe tissue necrosis and improve clinical outcomes.
